# Pulsed Thermal Method for Monitoring Cell Proliferation in Real-Time

**DOI:** 10.3390/s21072440

**Published:** 2021-04-01

**Authors:** Seppe Bormans, Gilles Oudebrouckx, Patrick Vandormael, Thijs Vandenryt, Patrick Wagner, Veerle Somers, Ronald Thoelen

**Affiliations:** 1 Institute for Materials Research (IMO), Hasselt University, 3500 Hasselt, Belgium; gilles.oudebrouckx@uhasselt.be (G.O.); thijs.vandenryt@uhasselt.be (T.V.); ronald.thoelen@uhasselt.be (R.T.); 2 IMEC vzw, Division IMOMEC, 3590 Diepenbeek, Belgium; 3 Biomedical Research Institute (BIOMED), School of Life Sciences, Hasselt University, 3500 Hasselt, Belgium; patrick.vandormael@uhasselt.be (P.V.); veerle.somers@uhasselt.be (V.S.); 4 Laboratory for Soft Matter and Biophysics, KU Leuven, 3001 Leuven, Belgium; patrickhermann.wagner@kuleuven.be

**Keywords:** biofilm, cell proliferation, *Saccharomyces cerevisiae*, pulsed thermal method

## Abstract

The study of cell proliferation is of great importance for medical and biological research, as well as for industrial applications. To render the proliferation process accurately over time, real-time cell proliferation assay methods are required. This work presents a novel real-time and label-free approach for monitoring cell proliferation by continuously measuring changes in thermal properties that occur at the sensor interface during the process. The sensor consists of a single planar resistive structure deposited on a thin foil substrate, integrated at the bottom of a cell culture reservoir. During measurement, the structure is excited with square wave current pulses. Meanwhile, the temperature-induced voltage change measured over the structure is used to derive variations in the number of cells at the interface. This principle is demonstrated first by performing cell sedimentation measurements to quantify the presence of cells at the sensor interface in the absence of cell growth. Later, cell proliferation experiments were performed, whereby parameters such as the available nutrient content and the cell starting concentration were modified. Results from these experiments show that the thermal-based sensor is able to accurately measure variations in the number of cells at the interface. Moreover, the influence of the modified parameters could be observed in the obtained proliferation curves. These findings highlight the potential for the presented thermal method to be incorporated in a standardized well plate format for high-throughput monitoring of cell proliferation.

## 1. Introduction

The study of cell proliferation plays an important role in many medical and biological research fields. Cell proliferation measurements can be used for diagnostic [1,2] and prognostic [3] purposes. Investigating the influence of certain compounds on the cell proliferation process is crucial to drug discovery research [4,5,6]. Furthermore, proliferation experiments contribute greatly to advances in the emerging field of regenerative medicine, such as tissue engineering [7,8]. These many useful applications generate a great demand for reliable high-throughput methods for monitoring cell proliferation.

At present, the most common way of assesing the growth in cell cultures is by visual examination using a microscope or spectrophotometry [9,10,11,12]. These methods require sampling a fixed volume from the cell suspension, which can be time-consuming. As the sample has to contain evenly distributed cells in suspension, it is extremely important to ensure that the culture is well mixed by shaking before taking a sample. A drawback of this technique is that it is an endpoint method, and thus only provides information on the proliferation process at a specific moment [13].

Another way of monitoring cell proliferation is by the use of metabolic assays. The colorimetric MTT (3-[4, 5-dimethylthiazol-2-yl]-2, 5 diphenyl tetrazolium bromide) assay is the most common example [14] and requires the addition of a marker. It is less elaborate than direct visual cell counting using a hemocytometer, and given that MTT assays are typically performed on 96-well plates, they can be performed at relatively high throughput. However, MTT assays do not allow for real-time monitoring of the proliferation process. Another drawback is that multiple studies have reported that various compounds can interfere with the conversion of the MTT dye to formazan, as is discussed in [15].

To render the cell proliferation most accurately over time, real-time sensing methods are required. An established example of such a method is electrical impedance spectroscopy (EIS) [16,17]. This label-free method relies on the difference in intrinsic electrical properties between cells and the culture medium. Therefore, the measured impedance will change as the number of cells increases. EIS electrodes can be integrated at the bottom of the culture reservoir. This enables real-time monitoring of the proliferation of adherent cells. Disadvantageously, the electrical fields generated during EIS measurements can damage the cells. Even low electrical fields of 60 V/cm can damage muscle and nerve cell membranes [18]. However, even lower electrical fields can cause changes in the membrane potential of cells, which can affect the activity of membrane channels [19,20]. Furthermore, EIS measurements can also be susceptible to signal drifts caused by electrochemical processes [21,22].

Alternatively, thermal sensors can be used for real-time monitoring of cell proliferation. Thermal-based methods do not expose the cells to electric fields and are unaffected by electrochemical processes. A thermal-based cell proliferation assay can be done on the basis of heat generated by the metabolic activity of cells [23,24,25]. Analogous to EIS, a thermal read-out can also be based on the difference in thermal properties of individual cells and the liquid growth medium they reside in [26]. This principle is demonstrated by Reyes et al. using a thermal read-out based on alternating current (AC) for monitoring biofilm formation [21]. This AC thermal method is often also called the 3ω method [27].

The aim of this work is to expand the available methods for real-time monitoring of cell proliferation by introducing an alternative thermal-based approach. With the presented method, a more simple pulsed direct current (DC) heating approach is used. This approach allows for easier sensor excitation and data interpretation as compared to AC heat transfer sensors that require complex hardware such as a lock-in amplifier. Furthermore, this pulsed approach can easily be multiplexed, which allows for multichannel measurements with only one read-out device.

The suggested pulsed measurement principle is derived from the Transient Plane Source (TPS) method [28,29]. The TPS method is a well known method for measuring the absolute thermal properties of various materials such as solids [29,30,31,32,33,34], fluids [35], powders [36] and thin films [37,38]. However, despite the wide use of this method, literature mentions no use of a pulsed method for dynamic biologically relevant monitoring applications. This work builds upon our earlier work in which we presented a proof of principle for measuring dynamic thin film thickness in real-time [39]. In this contribution, we highlight the applicability of this sensor for biological applications by performing cell proliferation experiments under varying conditions.

## 2. Working Principle

The presented thermal sensor aims to measure variations in the number of cells at the sensor interface. The intention of this work is to exploit this in order to monitor cell proliferation. The method relies on the difference in the thermal properties between the individual cells and the liquid medium they are growing in. This difference in thermal properties has been reported in previous studies [21,26]. More specifically, these studies have shown that the thermal conductivity (κ) of individual cells is lower than that of liquid culture media. Therefore, we assume that during proliferation, as the number of cells at the interface increases, this layer of cells will increasingly act as a thermal barrier between the sensor surface and the liquid medium.

**Figure 1:**
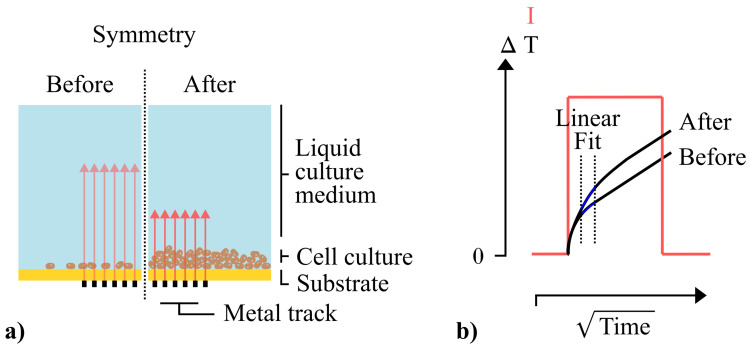
(**a**) Schematic cross-section of a planar resistive thermal sensor during measurements on a cell culture before and after proliferation. Heat transfer from the metal track to the liquid culture medium is illustrated by red arrows. We expect that a growing cell culture at the interface will impede the heat transfer. (**b**) As a result, when an identical Joule heating current pulse is applied, it is expected that the initial part of the temperature curve will become steeper as more cells accumulate at the interface. By monitoring the variation in the slope of this linear regression, we expect to be able to render cell proliferation in real-time.

The intention is to monitor this expected increase in thermal insulation at the sensor interface via a method derived from the Transient Plane Source (TPS) method [28,29]. The used sensor consists of a single planar metal structure that is deposited on a thin, flexible substrate, as shown in Figure 1a. The structure can be used as a heating element by applying a Joule heating current. Sequentially, the resulting temperature change in the metal structure itself can be evaluated by measuring the resulting change in electrical resistance of the metal structure and converting it to temperature using Equation (1) [28,29]. Both heating and temperature sensing can be performed sequentially by applying the 4-wire measurement principle by which a current is sourced and voltage is measured. For this, a Source Measure Unit (SMU) can be used.

(1) $$R=R_{ref}\left[1+\alpha \left(T-T_{ref}\right)\right]$$

where:

*R* = electrical resistance of structure [Ω]
*T* = temperature of structure [K]
$R_{ref}$ = electrical resistance at temperature $T_{ref}$
α = temperature coefficient of resistance [1/K].

The temperature response of the sensor will be determined by the ability of the sensor to dissipate heat. Hence, the temperature response will depend on the thermal properties of the material that it is in contact with [28,29]. As is done with conventional TPS measurements [28,29], the transient curves are plotted as a function of the square root of time (Figure 1b). Conventionally, linear regressions obtained at the latter linear part of the transient curves are used to determine the absolute thermal effusivity (*e*) of the sample that is being measured Equation (2).

As illustrated in Figure 1a, in this work, the temperature response will be influenced by both the liquid culture medium and the cell culture at the sensor interface. As the number of cells at the interface increases, we expect that the additional thermal insulation will result in a steeper temperature increase at the initial part of the curve (Figure 1b). This heat-blocking effect of thin films on pulsed thermal measurements has been observed before by Ahadi et al. as an undesired effect [37], and was exploited in our own earlier work on a thermal method for measuring dynamic thin film thickness [39]. In this present work, we will assess whether we can use this effect to monitor biological processes such as cell proliferation. We will monitor these changes in the interface over time by continuously applying thermal pulses and determining the slope of a linear regression within a fixed time interval at the start of the heat pulses (Figure 1b). Furthermore, we will evaluate whether we can relate changes in the slope to the number of cells at the interface.

(2) $$e=\sqrt{k\cdot \rho \cdot c_{p}}$$

where:

*e* = thermal effusivity [Ws^1/2^/m^2^K]
*k* = thermal conductivity [W/mK]
ρ = density [kg/m^3^]
$c_{p}$ = specific heat capacity [J/kgK].

When using this method, careful attention should be given to the selection of a suited time interval for the linear regression. The choice for the start time and end time of the regression interval will be determined by the required probing depth of the thermal pulses. To determine the start time, the intention is to find the exact time at which the probing depth of the applied thermal pulse exceeds the thickness of the substrate. The choice for the end time of the regression interval will be influenced by the thermal properties the cells and the thickness of the cell layer after proliferation. Note, however, that attention should also be given to ensuring that the linear regression in this interval has an adequate coefficient of determination (R^2^). In this work, the regression interval is determined experimentally, by proof of principle measurements on cell sedimentation. Later, slopes of linear regression within this time interval were closely monitored during cell proliferation experiments.

## 3. Materials and Methods

This section first describes the fabrication process of the developed sensor device as well as the overall measurement setup used during the experiments. Next, the procedures of the performed experiments are described. Initially, cell sedimentation experiments were performed to investigate the influence of the amount of cells on the temperature response of the sensor. Thereafter, cell proliferation experiments were carried out.

### 3.1. Measurement Setup

**Figure 2:**
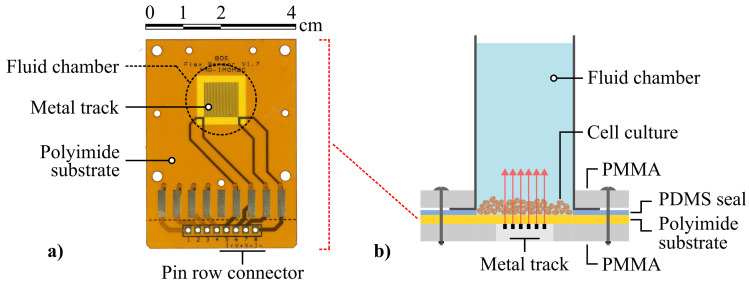
(**a**) An image of the sensor used for the detection of cells containing a metal track deposited on the polyimide substrate. (**b**) Schematic representation of the experimental setup during measurement of a cell layer. Heat pulses propagating from the heating structure through the cell layer and growth medium are represented by the red arrows.

The sensor used for the detection of cells is shown in Figure 2a. It is an in-house designed flexible heater containing a square meandering conductive track, with an overall size of 9 mm × 9 mm. The track consists of a copper layer, with a height, width, and spacing of respectively 15 µm, 100 µm, and 100 µm. The metal structure was deposited on a flexible polyimide substrate with a thickness of 40 µm. The temperature coefficient of the electrical resistance was determined experimentally (α = 3.818 × 10${}^{-3}$ ± 0.138 × 10${}^{-3}$ K${}^{-1}$). This value complies with values for the temperature coefficient of resistance of copper that can be found in literature [40]. At room temperature (R${}_{init}$, 20 °C), the initial electrical resistance of these structures ranges from 7 Ω to 10 Ω.

A liquid compartment was built around the flexible heater. Figure 2b shows a schematic illustration of the used experimental setup during measurement. A polymethyl methacrylate (PMMA) layer of 6 mm was used as a backplate for the sensor. It contains a cut-out of 9 mm × 9 mm directly below the meander structure to reduce energy loss to the environment. The flexible PCB is placed on this support with the copper facing down. This is done to prevent electrical shorts and corrosion of the copper due to the presence of liquid. The fluid chamber was made of a polypropylene cylindrical tube with an inner diameter of 19 mm and a maximum capacity of 25 mL. This allows for easy addition and removal of the solutions to be measured. A polydimethylsiloxane (PDMS) gasket with a thickness of 1 mm was placed between the liquid compartment and the heater-substrate to ensure a leak-free seal. This gasket features a hole with the same diameter as the fluid chamber. In order to hold the liquid compartment in place and generate enough pressure on the seal, another 6 mm PMMA layer was used in combination with four screws, to apply an evenly distributed pressure.

**Figure 3:**
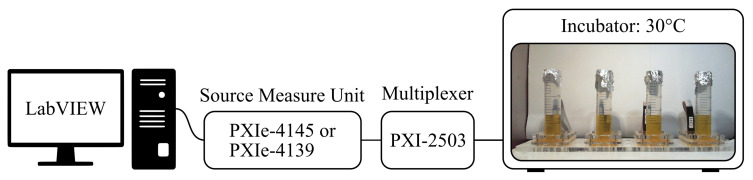
Schematic overview of the used measurement setup. Depending on the type of experiment, two different, yet similar, Source Measure Units were used. It should be stressed, though, that only one is required at a time.

Figure 3 depicts a schematic overview of the complete measurement setup used to perform the cell measurements. A Source Measure Unit (SMU) is used to drive the sensors. It generates heat pulses by applying a current to the sensor and reads back the applied voltage. This way, the electrical resistance of the structure is known at any given moment. Multiple channels can be measured sequentially by connecting the SMU to a multiplexer (MUX). As the applied heat pulses have to be uniform for all sensors, the same power needs to be applied. This is accomplished by taking the initial resistance value of the used sensor into account while calculating the required current. An in-house-designed LabVIEW program controls the measurement system.

### 3.2. Cell Sedimentation

To verify whether a varying amount of cells at the interface will influence the sensor temperature response, cell sedimentation experiments were performed. With sedimentation measurements, we can vary the number of cells at the sensor interface in a more controlled way as compared to proliferation measurements. Therefore, these measurements are appropriate to determine a suited regression interval for all later experiments on cell cultures. Furthermore, since no proliferation will occur during these experiments, we expect to measure no further changes in the slopes after the sedimentation process is complete. Therefore, we can use these tests to verify the sensor stability over time. Additionally, we can investigate the variation in measurement results between different sensors by repeating the sedimentation experiments with multiple sensor devices.

Cell suspensions were made of Dr. Oetker Dry Yeast (Bielefeld, Germany) in purified water (Sartorius). The stock solution of 8 mg/mL was diluted to concentrations of 4, 2, 1, 0.5, 0.25, and 0.125 mg/mL. Since no nutrients are added, we expect that no proliferation will occur. Therefore, we expect that after sedimentation, the number of cells at the sensor interface will vary with identical proportions. For comparison with a fully static sample, pure water (0 mg/mL) is also measured.

Before the start of the measurements, the fluid reservoirs were sterilized with 70 *v*/*v*% ethanol and blow-dried with nitrogen. Then, 1.5 mL of each well-shaken cell suspension was added to the fluid chambers and covered with a glass slide to reduce evaporation. The suspensions were left to sediment at room temperature for a total duration of 12 h. Meanwhile, thermal pulses were applied continuously. Each applied constant current pulse has a power of 0.5 W, and a duration of 1 s. A cooling time of 60 s was provided, which allows the sensors to cool down back to room temperature before each new pulse is applied. This implies that for the sedimentation experiments, the required time to obtain a value of the slope is 61 s. All sedimentation measurements were performed using a source measure unit (PXIe-4139, NI, Austin, TX, USA), measuring at 500 Hz during the heating step. The SMU is coupled with a multiplexer (PXIe-2503, NI). To investigate the reproducibility, this measurement procedure was repeated four times for each different cell suspension concentration. Hereby, for each measurement, a different sensor was used.

### 3.3. Cell Proliferation

Cell proliferation measurements were performed in real-time. To highlight the sensors’ ability to measure variations in these proliferation processes, two parameters were altered. The first parameter focussed on varying the D-glucose fermentable carbon source in the growth medium. Here, we expect the most growth to occur in the medium with the highest nutrient concentration present. The second parameter focussed on the starting concentration of yeast cells. As cells proliferate exponentially, growth will be observed the earliest under conditions with the highest starting concentration. To verify the proliferation process, Optical Density (OD${}_{600}$) measurements were performed in parallel. This was carried out by using multiple identical sensors, where the same starting concentration was used. At every sampling step, a sensor was removed from the incubator, shaken well, and diluted into a cuvette (Greiner Bio-One, Kremsmünster, Austria). A SmartSpec 3000 Spectrophotometer (BIO-RAD, Hercules, CA, USA) was used to determine the OD${}_{600}$ values.

A laboratory strain of *Saccharomyces cerevisiae*, BY4742, was used to perform the cell growth measurements. Before the start of each measurement, a preculture was grown by shaking overnight in YP (yeast extract–peptone) medium supplemented with 2% D-glucose in an incubator at 30 °C. Next, the sensors were sterilized with 70 *v*/*v*% ethanol and hot air-dried to avoid carryover of cells between experiments. A volume of 10 mL growth medium was added to the fluid compartment and supplemented with the antibiotic Ampicillin to prevent bacterial contamination. Finally, cells from the preculture were inoculated in the medium with the desired starting concentration, covered with aluminum foil and left to grow overnight in an incubator at 30 °C.

For the varying nutrient measurements, a stock solution of 10.00 g/L glucose was used and diluted down to 2.50 g/L and 0.16 g/L. These were then supplemented to the YP medium, and cells with a starting concentration of OD${}_{600}$ = 0.2 was used for all measurements. The varying starting density measurements used the same stock solution, but here the cells were inoculated with the following concentrations: OD${}_{600}$ = 0.1; OD${}_{600}$ = 0.2; OD${}_{600}$ = 0.4; OD${}_{600}$ = 0.8.

All proliferation measurements were performed using a PXIe-4145. It allows four channels to be measured sequentially. For all cell proliferation measurements presented below, thermal pulses were applied with a power of 0.5 W and a duration of 1 s. As *Saccharomyces cerevisiae* has a doubling time of approximately 90 min, it suffices to apply a pulse every 5 min. Therefore, the cooling time was increased to 300 s. This still allowed the sensor to cool down to room temperature without missing crucial proliferation information.

## 4. Results and Discussion

### 4.1. Cell Sedimentation

**Figure 4:**
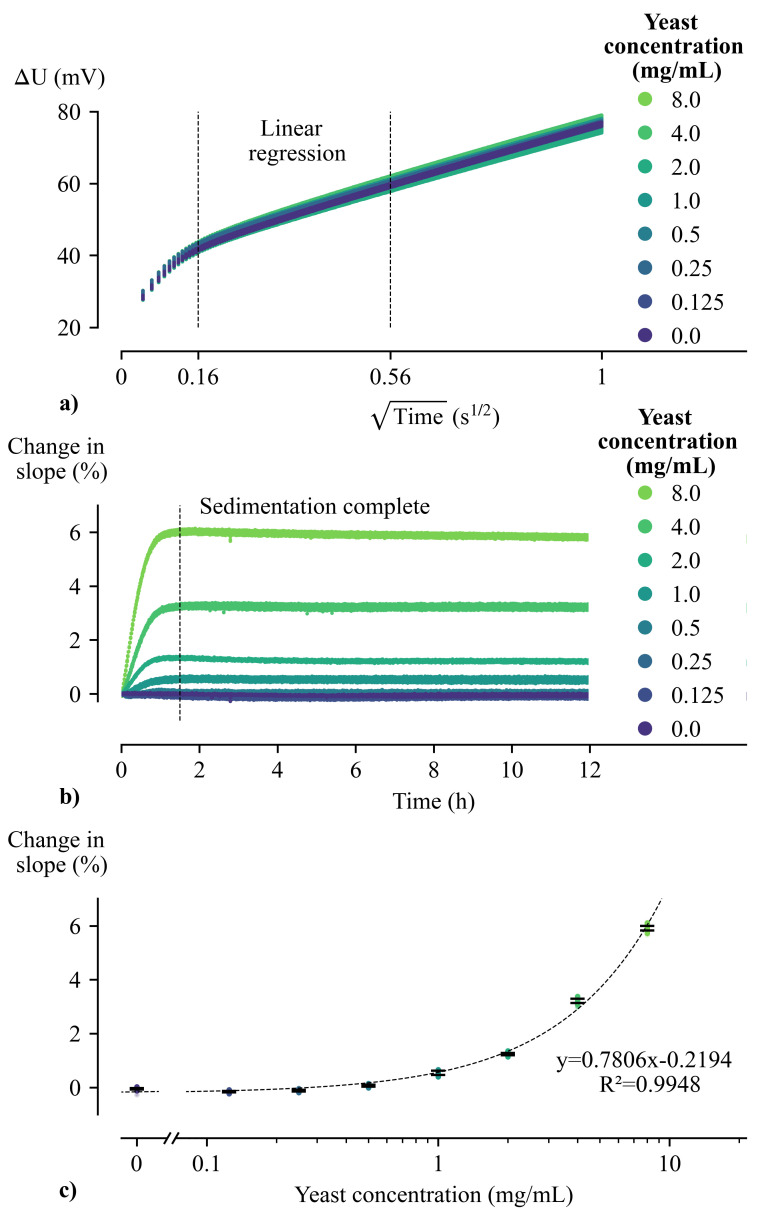
(**a**) Constant current pulses were applied to different sensors whilst various cell suspensions were left to sediment on the sensor surface. The resulting voltage changes measured over the metal structures are plotted on top of each other. It is expected that an increasing amount of cells at the sensor interface will result in a steeper slope within the selected linear regression interval. (**b**) The percentage change in slope is plotted as a function of time. For each concentration, this plot contains data of four unique sensors. It is clear that the slope of the sensor temperature response is influenced by the amount of cells at the interface. Within the first 1.5 h, cells accumulate at the sensor surface over time, and thereby cause an increase in slope. After the sedimentation is complete, the sensor signal remains stable over time for all sensors. (**c**) The sensor response after sedimentation, from 2 h to 12 h, is plotted as a function of the concentration of the added cell suspension (logarithmic scale). This plot illustrates the linearity of the sensor response to a varying amount of cells at the interface. Within the demonstrated range, the average standard deviation is 0.29 mg/mL.

In order to study the ability of the sensor to monitor the presence of cells, in the absence of cell growth, yeast cells were allowed to sediment on the sensor surface in the absence of nutrients. Different concentrations of dried yeast were resuspended in water, added to the fluid reservoirs of the sensors, and their sedimentation at the sensor surface was monitored by applying thermal pulses for a total duration of 12 h. Figure 4a shows all the changes in voltage measured with one sensor for each concentration during this period. Converted to temperature (1), these voltage changes of 80 mV correspond to a temperature change of approximately 6 K. The expected thermally insulating influence of the cells is not directly visible from these raw voltage curves. Therefore, closer data analysis in the form of linear regression is required to visualize small variations in the rate of the sensor temperature increase.

For the linear regression, the time interval between 25.6 ms, and 313.6 ms was chosen. This corresponds with the interval of [0.16, 0.56] s${}^{1/2}$ marked in Figure 4a. Within this interval, accurate linear regressions were performed on each voltage curve (R^2^ ≥ 0.98), and the observed slopes showed a good correlation with the amount of sedimented cells, as is discussed further.

In Figure 4b, the percentage change in slope, relative to the slope of the first applied pulse, is plotted as a function of time. Plotting the percentage change in slope allows us to visualize variations in slope over time whilst also eliminating the influence of differences in R*_init_* between sensors [39,41]. We presented this way of eliminating R${}_{init}$ in a previous work, [39]. During the first 1.5 h of the sedimentation process, an increasing number of cells at the sensor interface causes an increase in the slope of the temperature curves (Figure 4b). After that time, all cells have accumulated at the bottom of the fluid reservoir, and the slope no longer changes. Increasing the initial concentration of yeast cells led to a higher final percentage change in slope. The final percentage change in slope remains stable for all experiments for the total measurement duration of 12 h. This confirms that the sensors are stable over time, free of any drift.

Figure 4c shows the sensor response to varying amounts of cells at the interface. For this plot, the final percentage change of all experiments, i.e., all datapoints between 2 h and 12 h (Figure 4b), are plotted as a function of the concentration of the added cell suspension. For better visibility of the lower yeast concentrations, a logarithmic scale was used. The sensor response to the amount of cells can be approximated well by linear regression (y = 0.7806x − 0.2194), as indicated in Figure 4c. Within the demonstrated range of 0 mg/mL to 8 mg/mL, the sensor can determine the concentration of the cell suspension with an average standard deviation of 0.29 mg/mL. Since no significant difference is observed between sensors, we conclude that data of different sensors can be compared interchangeably without the requirement for additional sensor calibration steps.

By performing cell sedimentation experiments, it has been confirmed that the sensor is sensitive to varying amounts of cells at the interface. Moreover, the observed sensor stability, the linear sensor response, and the minimal required calibration suggest that these sensors can be well suited to monitoring cell proliferation. Therefore, in the following section, exploratory cell proliferation experiments will be reviewed. The data of all following experiments will be processed as described in this section. Thus, linear regressions on heating curves within the same time interval will be investigated.

### 4.2. Cell Proliferation

#### 4.2.1. Varying Concentrations of Nutrients

**Figure 5:**
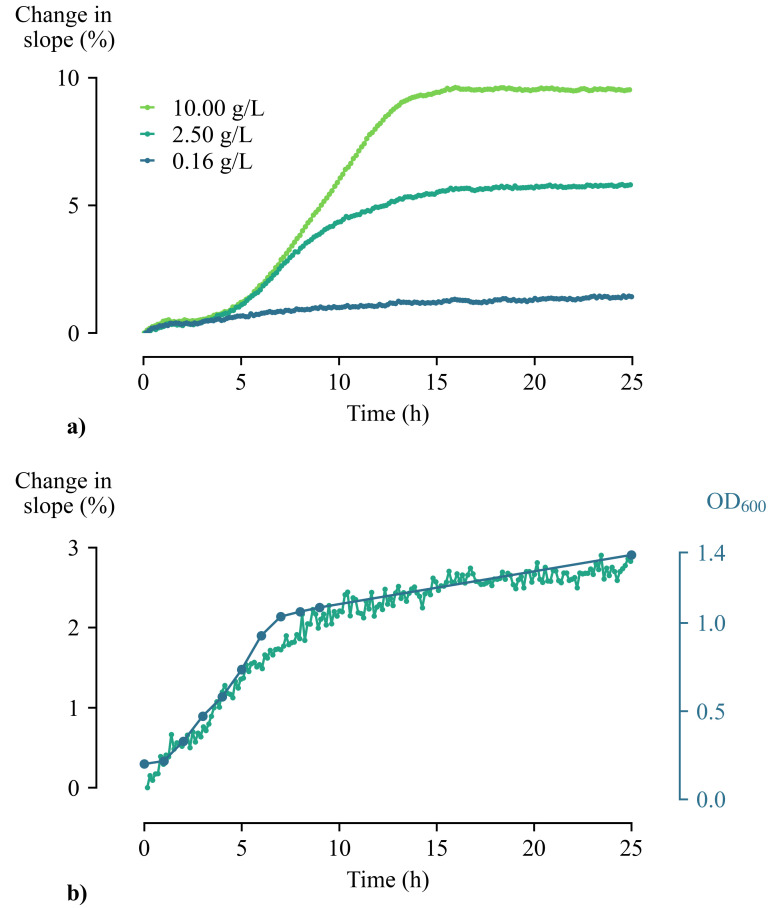
(**a**) Relative percentage change of slopes of *Saccharomyces cerevisiae* proliferated in growth medium with varying glucose concentration. It is clear that the slope of the sensor temperature response is influenced by the amount of available nutrients. (**b**) OD${}_{600}$ measurements (blue) were performed in parallel to verify the results from the thermal measurements (green).

Cells were inoculated in YP medium supplemented with 0.16, 2.50, and 10.00 g/L glucose, at a start OD${}_{600}$ of 0.2. Figure 5a) shows the percentage change in slope as a function of time for the three measured glucose concentrations. During the first 5 h of the proliferation process, the cells lie on the sensor surface and adjust to the environment. No major change in slopes is observed. After 5 h, the cells start to proliferate in a logarithmic manner and consume available nutrients. The more nutrients are present, the longer the cells will proliferate. This results in thicker cell layers at the interface of the sensor, impeding more heat transfer as they grow. A higher percentage change in slopes can therefore be observed. Once all nutrients are depleted, the proliferation process will stop, and the slopes no longer change.

In order to verify these results from thermal measurements, Optical Density (OD${}_{600}$) measurements were performed in parallel for the lowest concentration of glucose (=0.16 g/L), as described in Section 3.3. As these measurements require manual sampling, only 11 sample points were taken. During the first ten hours, one sample was taken each hour. Right before stopping the experiment, the last sample was obtained. The comparison of the optical density measurements with the measured thermal data is shown in Figure 5b). This clearly illustrates that the thermal data follow the pattern of the OD${}_{600}$ measurements, which confirms the hypothesis that growth is being measured.

#### 4.2.2. Varying Starting Concentration of Cells

**Figure 6:**
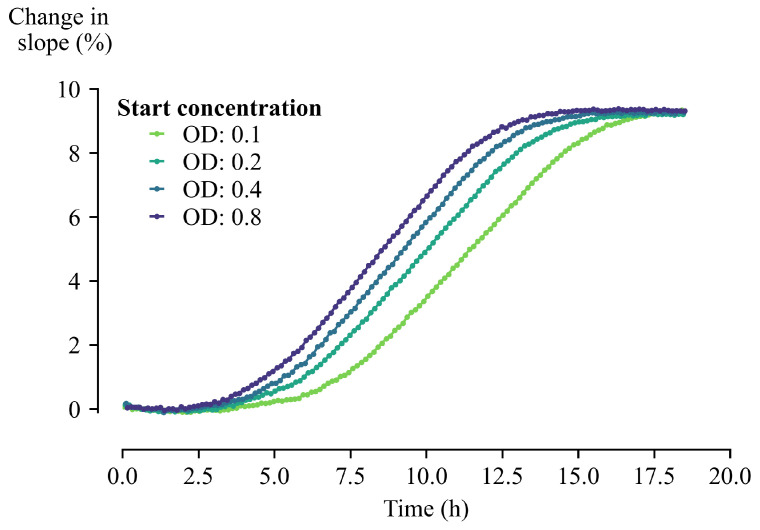
The percentage change in slope of proliferating *Saccharomyces cerevisiae* in YP medium with varying starting concentration. It can clearly be seen that these slopes are influenced by the amount of cells present at the beginning of the measurement.

Measurements with varying starting concentrations were performed, altering the amount of starting cells while keeping the glucose concentration of the growth medium constant (=10.00 g/L). As cells proliferate on the interface of the sensor, an increase in the slope of the temperature curves can be observed (Figure 6). The sensor containing the highest starting concentration will reach the exponential growth phase the fastest, resulting in an earlier change in slopes compared to the other sensors. A serial dilution of the starting concentration systematically delays this observation. Since the growth medium was the same for all conditions in the stationary growth phase, approximately equally thick layers were formed on the sensor interface. This can be observed as the relative changes in the normalized slopes are equal at the end of the measurement.

The evidence presented in this section suggests that this thermal method can be used for monitoring cell proliferation in real-time. The accuracy of the sensors is sufficient to register differences between proliferation parameters such as the available nutrient content and the cell starting concentration. This current work is limited to experiments on suspension cells. However, based on our own earlier work on thermal measurements on thin films, the principle should also be applicable to adherent cells [39]. However, it should be noted that the heat blocking effect of a single monolayer of adherent cells is most probably significantly smaller as compared to that of a suspension cell culture.

## 5. Conclusions

This contribution presents a novel, real-time method for monitoring the number of cells at the bottom of a cell culture reservoir. In the past, pulsed thermal methods have been widely used for determining the thermal properties of various static samples. In this work, however, thermal pulse excitation is used to measure dynamic variations in thermal properties near the sensor interface. These thermal variations can be related directly to the amount of biomass present. This method makes use of a sensor design that consists of a single planar metal structure deposited on a thin foil substrate. Where previously reported thermal-based cell proliferation monitoring methods utilized an alternating current (AC) sensor actuation, direct current (DC) pulses are used in this work. This offers a significant simplification of the required hardware and data interpretation. For the read-out, a single Source Measure Unit (SMU) is required to perform a 4-wire measurement. Optionally, a multiplexer can be used to perform the measurement sequentially on different sensors for increased throughput. For the data interpretation, simple linear regressions need to be performed on the sensor temperature response curves. The presented thermal method can accurately render the proliferation process over time without being affected by electrochemical changes in the media due to bacterial growth. The risk of disturbing the proliferation process due to the exposure of cells to electric fields is eliminated, as the sensor structure is electrically insulated.

First, the working principle was demonstrated by performing cell sedimentation measurements using cell suspensions of different concentrations. Hereby, the presented thermal sensor was able to monitor the sedimentation process over time and distinguish between the different concentrations. The results indicated that, within the tested range, the sensor responds linearly to a varying amount of cells at the interface. Furthermore, these measurements showed a high stability, and reproducibility between different sensors without the need for additional calibration steps.

Later, measurements were performed on cell cultures in an effort to monitor the proliferation process. The growth curves obtained from the thermal data proved to be comparable to curves measured via the optical density (OD${}_{600}$) measurement. Furthermore, the influence of variations in the starting concentration of cells or the nutrient content is clearly visible in the growth curves. These results clearly highlight the ability of the sensor to monitor the cell proliferation process over time. This current work is limited to proliferation experiments on suspension cell cultures. Principally it is also possible to apply this measurement principle to adherent cells. However, presumably, the heat blocking effect of a monolayer of adherent cells is much lower as compared to a suspension cell culture.

Given that the sensor is integrated at the bottom of the cell culture reservoir, the results indicate that this method might be useful for other biomass formation monitoring applications, such as measuring biofilm formation or investigating the influence of specific antibiotics on biofilm formation. The simplicity of the required hardware and data processing enables such applications to be explored at a high throughput. It should be noted that the current prototype sensor device is developed for mL-sized samples, whereas common cell proliferation assay methods are developed for a 96-well plate format that uses µL-sized samples. However, the sensor allows easy miniaturization and incorporation in standardized well plate formats.

## Data Availability

The data that support the results of this study are availble from the corresponding author, S.B., upon reasonable request.
